# Decision-making Factors Toward the Adoption of Smart Home Sensors by Older Adults in Singapore: Mixed Methods Study

**DOI:** 10.2196/34239

**Published:** 2022-06-24

**Authors:** Yuanyuan Cao, Mojisola Erdt, Caroline Robert, Nurhazimah Binte Naharudin, Shan Qi Lee, Yin-Leng Theng

**Affiliations:** 1 Centre for Healthy and Sustainable Cities Wee Kim Wee School of Communication and Information Nanyang Technological University Singapore Singapore; 2 Institute for Infocomm Research, Agency for Science, Technology and Research (A*STAR) Singapore Singapore

**Keywords:** aging in place, health care systems and management, telehealth, assistive technology, assisted living facilities

## Abstract

**Background:**

An increasing aging population has become a pressing problem in many countries. Smart systems and intelligent technologies support aging in place, thereby alleviating the strain on health care systems.

**Objective:**

This study aims to identify decision-making factors involved in the adoption of smart home sensors (SHS) by older adults in Singapore.

**Methods:**

The study involved 3 phases: as an intervention, SHS were installed in older adults’ homes (N=42) for 4 to 5 weeks; in-depth semistructured interviews were conducted with 18 older adults, 2 center managers, 1 family caregiver, and 1 volunteer to understand the factors involved in the decision-making process toward adoption of SHS; and follow-up feedback was collected from 42 older adult participants to understand the reasons for adopting or not adopting SHS.

**Results:**

Of the 42 participants, 31 (74%) adopted SHS after the intervention, whereas 11 (26%) did not adopt SHS. The reasons for not adopting SHS ranged from privacy concerns to a lack of family support. Some participants did not fully understand SHS functionality and did not perceive the benefits of using SHS. From the interviews, we found that the decision-making process toward the adoption of SHS technology involved intrinsic factors, such as understanding the technology and perceiving its usefulness and benefits, and more extrinsic factors, such as considering affordability and care support from the community.

**Conclusions:**

We found that training and a strong support ecosystem could empower older adults in their decision to adopt technology. We advise the consideration of human values and involvement of older adults in the design process to build user-centric assistive technology.

## Introduction

In Singapore, an aging population is on a rapid increase, and approximately 25% of Singaporeans will be aged ≥65 years by 2030 [1], posing a strain on hospitals and nursing homes. To ease this growing demand, the Singapore government has continuously enhanced community-based homecare and day care services, allowing older adults to *age in place*.

### Background

Smart home devices have been shown to reduce anxiety surrounding an emergency and have helped improve the confidence of older adults living alone [2]. Acknowledging this, the Singapore Housing and Development Board (HDB) started the Smart Enabled Homes initiative by encouraging the installation of smart home devices and apps in senior-friendly HDB studios [3]. The Singapore Ministry of Health also supports older adults in living independently by facilitating a wide range of social and community support services, including senior activity centers (SACs) [1]. SACs are drop-in centers often located in HDB rental blocks, where residents living in the same block or surrounding blocks are supported by affordable or free activities held by SACs. SACs also provide support services for frail or homebound older adults.

Many countries have demonstrated the demand and trend in adopting smart home devices for older adults [4-6]; however, adoption of smart home technologies among older adults remains low because of many factors [7]. In a recent scoping review, Astell et al [8] confirmed that assistive technologies were often viewed as a blatant indicator of aging so that older adults resisted the use of these technologies. This perspective strongly impacted the adoption of assistive technologies by older adults, whereby older adults using these technologies were tagged as being *old*, *lonely*, or *frail* [9]. This scoping review also highlighted older adults’ desire to depict their identity as consistent with independence, self-reliance, and competence.

Studies have found that perceived benefits or usefulness are the most critical motivational factors for accepting technology (eg, internet) by older adults [10]. The Technology Acceptance Model (TAM) [11] has been widely recognized and adopted as a tool to measure the acceptance of technology. The TAM proposes 2 key variables—*perceived usefulness* and *perceived ease of use*—to determine the use and acceptance of technology. To extend this to the older population, Chen and Chan [9] proposed a senior TAM that captures aspects such as computer self-efficacy and age-related cognitive and physical changes. To understand older adults’ behaviors in using the internet in China, Pan and Jordan-Marsh [12] expanded the TAM model to include 2 additional variables—*subjective norm* and *facilitating conditions*—which highlighted the importance of policy making in alleviating social and cultural obstacles facing older adults.

Technology acceptance and intention to use and adopt technology by older adults have been measured in many studies [12-15]. However, decision-making factors leading to the adoption of technology by older adults have not yet been exhaustively researched by any studies, not only by Astell et al [8]. Davenport et al [16] proposed a decision tree model comprising potential barriers to and facilitators of smart technology that requires decision processes by older adults. However, neither a perceived need for, acceptance of, nor intention to use technology by older adults necessarily leads to a decision to adopt technology or an actual adoption of technology. Thus, a gap remains in thoroughly understanding the decision-making process of older adults beyond their perceived acceptance of technology up to the point of their use and adoption of technology.

### Objectives

This study aimed to bridge this gap by exploring the in-depth decision-making factors leading to the adoption of technology by older adults. In particular, we introduced and installed smart home sensors (SHS) for older adults in Singapore and investigated their decision-making process until full adoption (postintervention installation and use) of SHS in their homes.

## Methods

### Ethics Approval

This study was approved by the institutional review board of Nanyang Technological University (NTU; IRB-2017-12-003, IRB-2018-01-002, and IRB-2019-04-030), Singapore. We collaborated with a commercial service provider partnered with HDB to provide SHS for this study. We recruited older adults from an SAC in Singapore, Adventist Home for the Elders (AHE).

### Study Design

**Figure 1:**
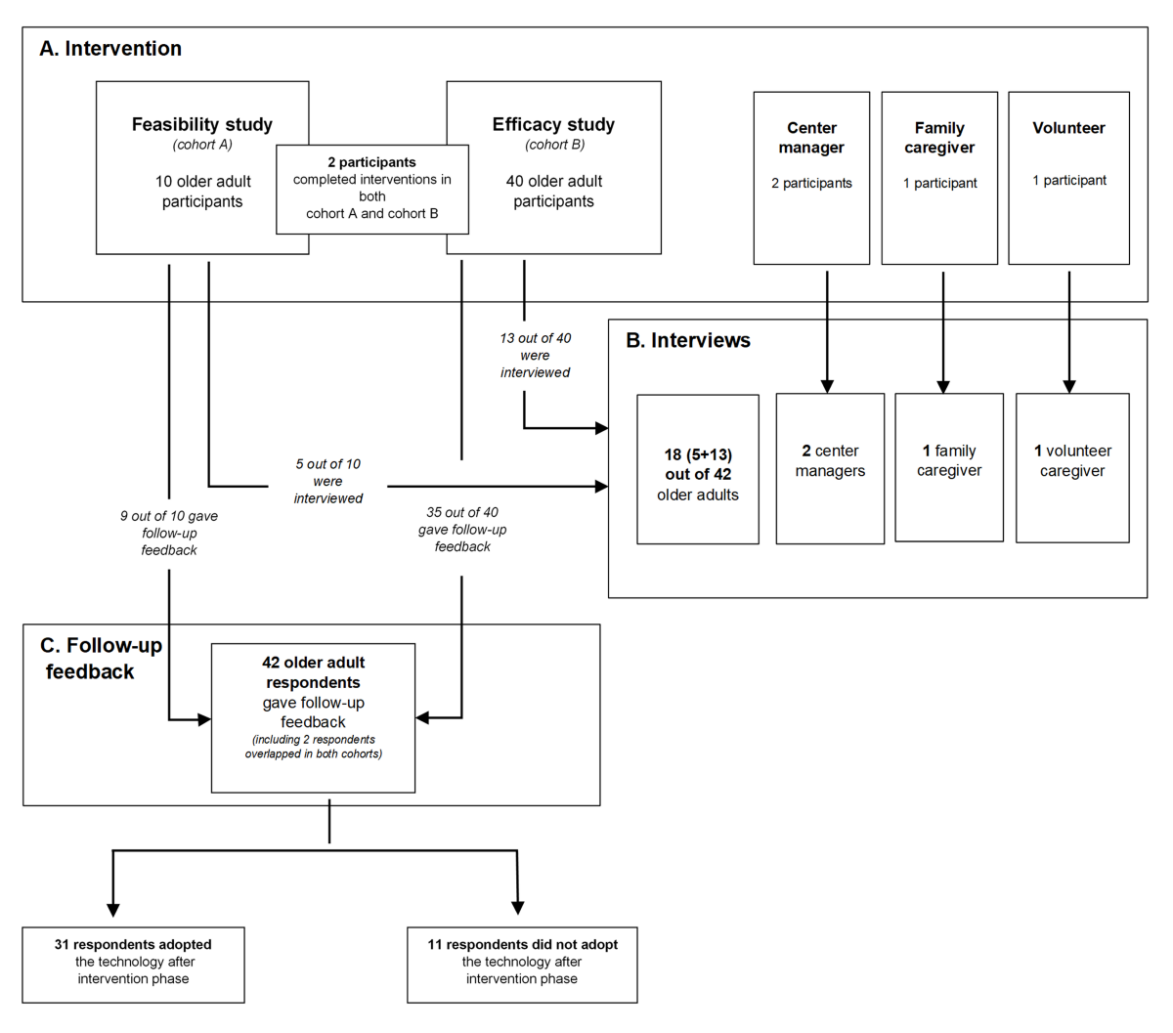
Overview of the study design and study participants. The study comprised 3 phases. Phase A is the intervention phase consisting of the feasibility study and the efficacy study (A). Participants involved in both studies are shown with overlapping participants depicted. Phase B is the interview phase, and the participants involved from the preceding phase A are depicted (B). Phase C is the follow-up feedback, and participants come from the feasibility study and from the efficacy study in phase A (C).

To gain a deeper understanding of the decision-making process of older adults toward the adoption of SHS, this study comprised 3 phases (Figure 1).

#### Intervention

The intervention comprised 2 parts: a feasibility study (cohort A) conducted between April and May 2018 and an efficacy study (cohort B) conducted between October 2018 and March 2019. The feasibility study aimed to assess the study design, tools, and technology systems with a small group of participants in preparation for the subsequent larger efficacy study. As an intervention, SHS were installed in the HDB flats of older adults for 4 to 5 weeks. The SHS technology comprised a bedroom assistant, 3 motion sensors, a smart plug, a door contact, a key tag, and a mobile app. Motion sensor, strategically placed in selected rooms (kitchen, bathroom, and living room), are considered to have higher chances of detecting emergency or fall cases. These sensors detect movement activities without capturing images. The smart plug shows appliance use (eg, which appliances are currently in use based on the amount of electricity consumed). Door contact can sense the opening and closing of the main entrance door. The key tag monitors the in-home and out-of-home statuses. The bedroom assistant comprises motion and sound sensors that detect irregular noise or inactivity in the bedroom. The sensors were linked to a mobile app to provide notifications to designated caregivers or next of kin in case of an emergency. The mobile app can also be used to monitor care recipients’ daily activities, receive intelligent notifications if something unusual occurs, and make calls for 24/7 personal assistance. On pressing an emergency button in the bedroom, an alarm is sent via the mobile app and as an SMS text message. After the intervention period, the AHE offered to waive subscription fees for SHS for a period of 2 years for all older adult residents of their HDB blocks, including the participants of this study.

#### Interviews

With maximum variation sampling [17], interviews were conducted with multiple stakeholders (n=22, including 18 older adults [users of SHS], 2 SAC center managers, 1 family caregiver [predefined SHS contact person], and 1 older adult volunteer). In addition to family caregivers, center managers and an older adult volunteer played the role of caregivers in the study. This extended caregiver role was designed based on a typical SAC setting in Singapore, where older adults living in the same HDB building are good neighbors who take care of each other. In addition, center managers are well trusted and provide support to the older adults daily.

#### Follow-up Feedback

Feedback from older adults (N=42) was collected after the intervention to understand the reasons for the adoption of or not adopting SHS (ie, subscribing or not subscribing to SHS after the intervention). Questions also included their decision to continue subscription beyond 2 years and the subscription fees they would be able to afford.

### Sampling and Recruitment

With the assistance of center managers at the AHE, we did purposeful sampling to recruit older adults for the study. In particular, inclusion criteria for the recruitment of older adults for the intervention were that they (1) were aged ≥55 years; (2) had voluntarily consented to take part in the study; (3) were able to communicate and express themselves clearly; and (4) were living alone or had the necessity of using SHS; furthermore, for interviews and follow-up feedback, that they (5) had taken part in the SHS intervention for at least 4 weeks.

We found that 10 older adults from cohort A and 40 from cohort B were eligible for recruitment for interviews and follow-up feedback (with 2 belonging to both cohorts). In total, 42 older adults consented to participate in the follow-up feedback. Overall, 2 participants did not provide consent, 3 were hospitalized, and 1 no longer resided in the AHE (Figure 1). Using purposeful sampling, we selected 43% (18/42) of participants for in-depth interviews (5 from cohort A and 13 from cohort B). We strived to represent different ethnicities in the community to reflect the Singapore context using maximum variation sampling [17]. We conducted interviews in Chinese (n=9), Malay (n=3), and English (n=6). In addition to conducting interviews with 18 older adults, 2 center managers, 1 family caregiver, and 1 volunteer were interviewed in English, with the aim of gaining a holistic perspective in the decision-making process. To understand the reasons for adoption or not adopting SHS after the intervention, follow-up feedback was collected from older adult participants who had completed at least 4 weeks of intervention.

### Data Collection and Analysis

Semistructured interviews were administered to participants directly after the intervention in May 2018 for cohort A and in April 2019 for cohort B. Interviews were based on a retrospective perspective on the timing of the intervention proposed by Sekhon et al [18]. Before the interviews, interview guidelines were developed for different study participant types (ie, older adults, family caregivers, and volunteers) based on 7 constructs of the theoretical framework of acceptability, including affective attitude, burden, intervention, coherence, ethicality, opportunity costs, perceived effectiveness, and self-efficacy [18]. Interview questions (Multimedia Appendix 1) were designed to elicit perceptions of general feelings, usefulness, satisfaction, effectiveness, convenience, intentions of subscription, and other concerns of older adults toward the service. *Service* mentioned in interviews referred to SHS. For example, 1 open-ended question was, “How did this service benefit you? Would you please share some details with us?”

Trained and experienced interviewers conducted semistructured interviews with participants at their preferred locations, mostly at their homes, to make them feel comfortable with the interview. On the basis of their experience, the interviewers would rephrase questions when they felt that the participants did not understand them initially. They would also probe deeper when they felt that participants had more to share about their experiences. We anonymized the identities of all participants, giving each a code from E001 to E042. The interview guidelines were transcribed into Chinese and Malay by 3 interviewers before the interviews. All interviews were transcribed and translated (9 from Chinese to English and 3 from Malay to English) and classified based on the type of study participant. A data-driven inductive approach was chosen to conduct a thematic analysis of the 22 transcripts [19,20]. At the start of coding, 3 researchers (YC, SQL, and NBN) individually conducted preliminary scanning of all transcripts and separately came up with a first draft of the coding scheme. Each transcript was carefully read and relevant words, sentences, and sections were identified as meaningful units of text and labeled with codes using the open code approach. Through an iterative process of comparing coded transcripts, we discussed our thoughts on code and subcode designations. We agreed to delete redundant themes, combined themes with similar meanings, and added new themes that might have been missed in others’ coding schemes. Thereafter, a common coding scheme is refined based on the definition of each code. We then separately coded transcript 1 using a refined coding scheme to strike the required credibility and reliability [21,22]. Next, we reviewed coding accuracy and consistency and discussed discrepancies. Following this, we achieved a final consensus on the coding scheme and felt intercoder reliability was reached. The remaining transcripts, 2-22, were equally shared and coded following the agreed coding scheme. Thereafter, codes were categorized to form themes and subthemes after discussions among the 3 coders (Multimedia Appendix 2).

As we wanted to collect feedback postintervention from all 42 participants regarding adopting or not adopting SHS, we decided to create a simple self-report questionnaire using a 5-point Likert scale. On the basis of preliminary data analysis of transcripts, we developed questions regarding reasons for adopting or not adopting SHS, depending on whether participants continued with SHS subscription. Open-ended questions were included to gather further details (Multimedia Appendix 3). Follow-up feedback was collected from all the 42 participants in May 2019.

## Results

### Overview

The demographics of the 42 study participants are presented in Table 1. Most participants were female (28/42, 67%) and of Chinese ethnicity (35/42, 83%), and 60% (25/42) had a primary education level or no formal education. Overall, 52% (22/42) of the participants had family support, although the majority lived alone (33/42, 79%). A total of 60% (25/42) of participants had above-average to excellent health status. The detailed demographic information is presented in Table 1.

**Table 1:** Demographics of older adult participants (N=42).

Measurements				Value
Age (years), mean (SD)				71.07 (6.46)
**Gender, n (%)**				
	Male			14 (33)
	Female			28 (67)
**Ethnicity, n (%)**				
	Chinese			35 (83)
	Malay			6 (14)
	Indian			1 (2)
**Education level,** **n** **(%)**				
	No formal education			6 (14)
	Primary			19 (45)
	Secondary			14 (33)
	Preuniversity			2 (5)
	University			1 (2)
**Living arrangement, n (%)**				
	Alone			33 (79)
	With family			8 (19)
**Family support, n** **(%)**				
	Without support			20 (48)
	**With support**			22 (52)
		From children	15 (68)	
		From spouse	4 (18)	
		From siblings	2 (9)	
		From other relatives (eg, niece)	1 (5)	
**Health status, n** **(%)**				
	Very poor			2 (5)
	Below average			5 (12)
	Average			10 (24)
	Above average			19 (45)
	Excellent			6 (14)

Figure 1 shows an overview of the study design and study participants. From the follow-up feedback, we found that 74% (31/42) of participants adopted SHS after the intervention, whereas 26% (11/42) of participants did not. Among those who subscribed to SHS after the intervention, from responses to open-ended questions, approximately 29% (9/31) were not sure about the duration of subscription they would continue with. For those who did not subscribe to SHS after the intervention, most (9/11, 82%) were not sure about the reasons, and only a few explicitly expressed reasons *not interested* or *afraid of being monitored*. Multimedia Appendix 3 provides details on the price, reasons, and duration of SHS subscription.

The interview participants comprised 18 older adults of different ethnicities (15 Chinese and 3 Malay). Overall, of the 18 participants, 13 (72%) were female and 5 (28%) were male; 2 (11%) center managers, 1 (6%) family caregiver, and 1 (6%) older adult volunteer were female of Chinese ethnicity.

**Figure 2:**
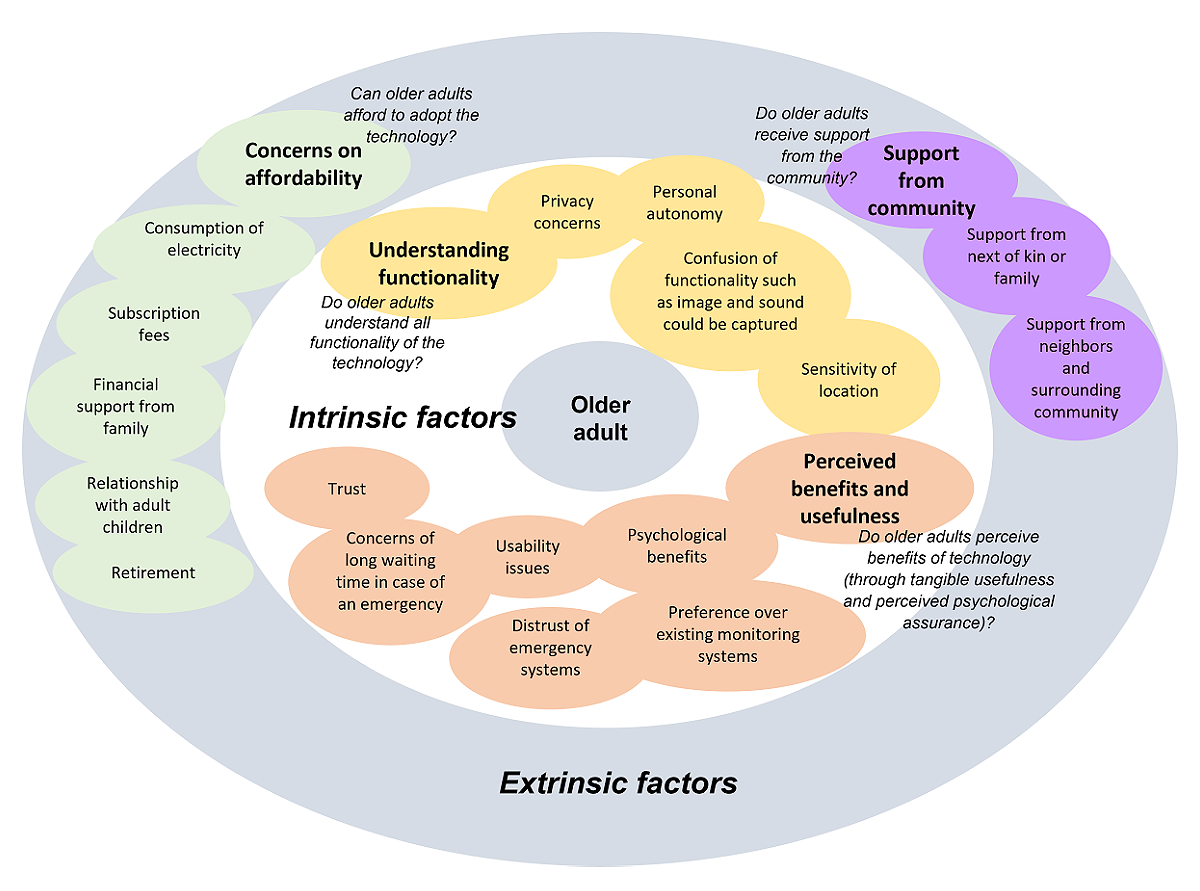
Decision-making factors toward smart home sensor adoption by older adults.

A total of 4 themes emerged from the interview results. Further analysis of the themes led to the decision-making factors shown in Figure 2. We elaborate on each theme and the resulting decision-making factors in the following sections.

### Theme 1: Understanding SHS Functionality

Understanding technology is an important prerequisite for its adoption. However, this is a challenge, particularly for older adults. Misunderstanding functionality can cause many concerns, especially in the initial stages when users get to know and use the technology for the first time. From interviews, we see that, initially, participants resisted using SHS as they did not understand the technology. In general, older participants took approximately 2 weeks to familiarize themselves with SHS and gradually felt comfortable with it. This demonstrated that understanding technology requires time. However, without a clear explanation of the technology, misunderstandings could still arise, even when given sufficient time.

Misunderstanding SHS has resulted in concerns about the intrusion of privacy. In our study, although it was explained that neither video cameras nor images were captured, older adults still felt stressed about having SHS installed in their homes. Older adults understood that motion sensors could detect their movements; however, they were unsure if they captured their images as well. In the first week, some participants expressed that they felt:

It is watching and capturing my every action.

E014, female, aged 67 years

I thought it captured my action and speech.

E023, female, aged 73 years

Another concern stemmed from the misunderstanding of the emergency alert functionality. Some older adults showed pessimism about receiving a response during an emergency. They found that if they and their next of kin did not understand the SHS emergency alert functionality, they could rely on receiving help in case of an emergency. Some asked:

How I can be responded [to] if anything happens to me.

E024, female, aged 64 years

On the one hand, this misunderstanding of the SHS functionality led some participants to avoid passing by or touching the sensors, as they were concerned that this would mistakenly alert their next of kin. On the other hand, 1 participant felt a false sense of security as she thought she could trigger an alert at any time by touching the sensors everywhere in her house, so she expressed:

It is really very convenient for me in a way.

E008, female, aged 70 years

Owing to uncertainty and confusion regarding SHS, 1 older adult pressed the emergency button to test whether it was really working. He was excited when he received a call from his family member, who was designated a contact person.

This shows that an explicit explanation is needed on how SHS detects movement and how the contact person is notified. This was also demonstrated when a few older adults who initially did not understand the SHS and felt it was intrusive changed their minds after the SHS service provider showed them how data were captured on the laptop. The participants then started to understand the functionality better. One older adult said that he felt safer after seeing the dashboard on a laptop:

Because it’s all by written or like captured like... they view they show me all the graph lah.

E028, male, aged 75 years

Appreciation for SHS rose when older adults understood the functionality. They expressed:

Some people don’t understand. They thought it was an infringement of their privacy, but actually it is not. I told them it is not, so I feel that this sensor is very good.

E008, female, aged 70 years

Some participants showed a great interest in learning innovative things and expressed the need for more knowledge about SHS. They were not fully satisfied with brief explanations, such as *sensors are for your safety*. They were eager to learn about SHS in greater detail. They shared:

Like [explaining to us], what is the purpose of installing one in the bedroom, why one in the kitchen and one in the washroom etc. Ah... like they can have a presentation at the center downstairs, project the sensor onto the screen and tell us “ah the reason to have one sensor installed in the kitchen is so that it can detect leaked gas” or something along those lines.

E036, male, aged 66 years

From the follow-up feedback, 36% (4/11) of those who did not adopt the SHS after the intervention did not understand how the system worked, which was not the reason why they decided not to subscribe. This means that there must be other factors that influenced their decision to not adopt SHS. An adequate understanding may not lead to the adoption of technology directly; nevertheless, it may lead to other factors in decision-making, such as the perceived benefits or usefulness of the technology.

### Theme 2: Perceived Benefits and Usefulness of SHS

#### Overview

**Figure 3:**
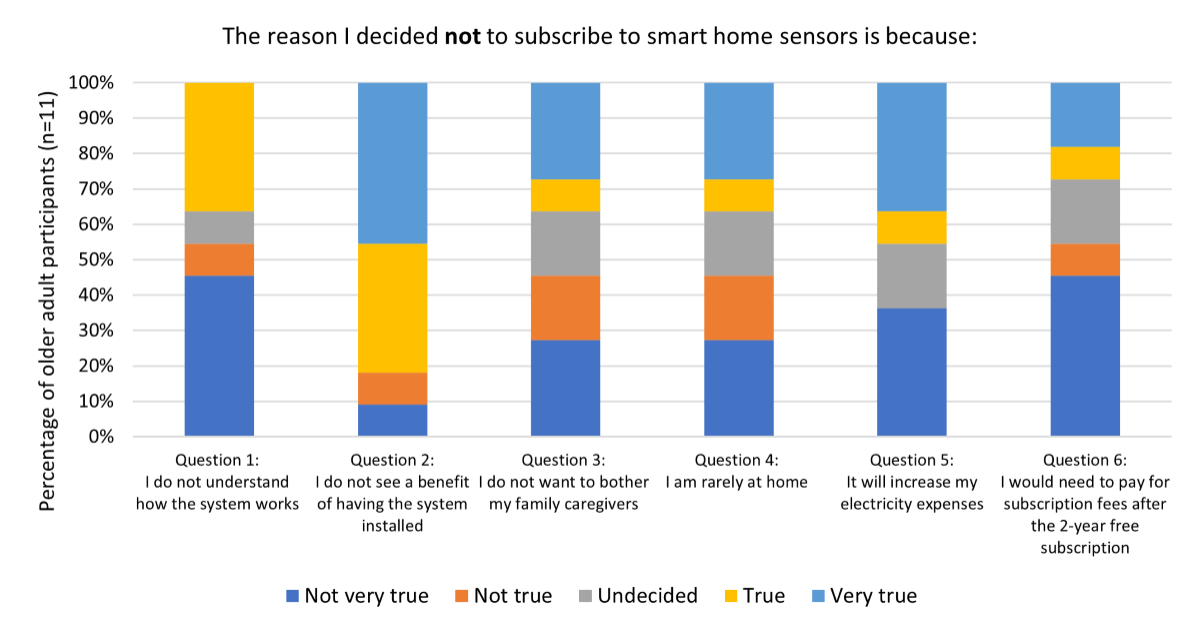
Reasons for not subscribing to SHS after the intervention (n=11). SHS: smart home sensors.

The perceived benefits and usefulness of SHS include psychological benefits and advantages of using SHS over other monitoring systems that older adults had experienced in the past. Figure 3 shows the reasons why the participants decided not to adopt the SHS. The most salient reason was not seeing a benefit in having SHS installed in their homes (Question 2: 9/11, 82% indicated *true* or *very true*).

#### Subtheme 2.1: Psychological Benefits

Assurance and a sense of security were the 2 main psychological benefits perceived from using SHS. Rather than mentioning specific tangible benefits, older adults said that the SHS made them feel at ease. Their feelings of security were mainly based on their trust in technology, that it will help them in case of an emergency. Some summarized psychological benefits as *easier* and *convenient* for them to remain safe. For example, with the emergency button, older adults said that they felt they would receive a response in case of an emergency and would be attended to immediately. One older adult said:

If something occurs, let’s say something occurs while sleeping, all I need to do is just pressing.

E039, male, aged 74 years

**Figure 4:**
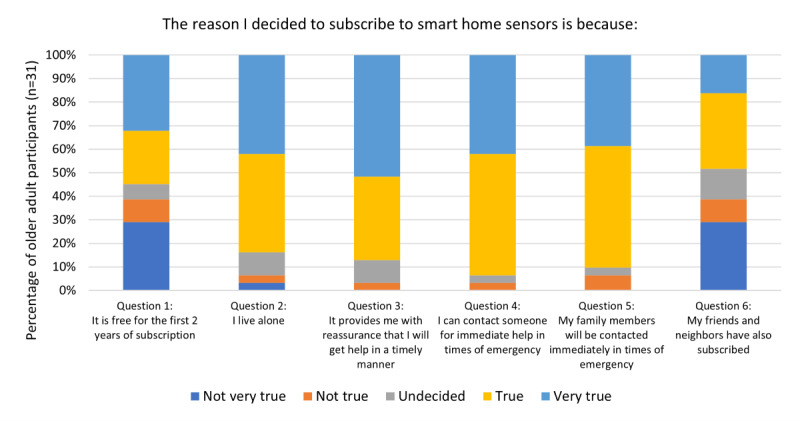
Reasons for subscribing to SHS after the intervention (n=31). SHS: smart home sensors.

From the follow-up feedback, of the 42 participants, we found 31 (87%) older adults subscribed to SHS after the intervention (see Figure 4 for the reasons). The most salient reason was because someone would be contacted for help in case of an emergency (Question 4: 29/31, 94% indicated *true* or *very true*). In turn, family members would be notified when there is an emergency (Question 5: 28/31, 90% indicated *true* or *very true*), and as such, these reasons provided participants with a sense of reassurance that they would get help in a timely manner (Question 3: 27/31, 87% indicated *true* or *very true*), especially so if they live alone (Question 2: 26/31, 84% indicated *true* or *very true*).

Some older adults’ perceived usefulness of SHS lies in receiving a quick response in case of emergency. For example, 1 older adult pressed the emergency button as a test. He felt that the system was very useful when his nephew called to check on him.

One older adult shared that she felt that SHS was helpful as her goddaughter had told her that she could see her movements via the mobile app. Another older adult, whose spouse used SHS, shared that it was useful because she could monitor her spouse’s movements. These comments demonstrate that positive feedback from caregivers regarding the usefulness of SHS can provide confidence to older adults. One older adult shared that his nephew:

Can see where I am, my activity in a particular hour, whether it is in the living room or bedroom or anywhere in the house... It’s useful. If I am not at home, he will know too.

E031, male, aged 73 years

Older adults were also motivated by neighbors’ experiences of using SHS:

I know because my neighbor the other time like in early 2018 [possibly Feasibility study], I think she already get I think like oh okay this is good actually [...] She already have it so I know what is actually happening [Laughter] [...] I’m aware of it long time ago.

E028, male, aged 85 years

On the one hand, older adults find SHS nonintrusive, as it is integrated into their daily lives and is only noticed in an emergency. On the other hand, this can lead to a feeling of indifference toward SHS or that *it is useless*, which could downplay its real value. Some shared that it did not bring much difference to their lives:

Nothing to disturb me, to attract our attention or whatever.

E024, female, aged 64 years

#### Subtheme 2.2: Preference of SHS Over Existing Monitoring Systems

Older adults expressed their preference for SHS compared with pull alarms already installed in their apartments. The reason is, in an emergency, older adults need to pull their cords to alarm their neighbors. Compared with this, older adults preferred SHS as being more convenient to use, as they preferred having to press a button rather than pulling a cord.

In addition, older adults found SHS less intrusive compared with video surveillance systems. A family caregiver shared:

It’s better than those pinhole surveillance cameras, as it does not...it protects our privacy.

FC001, female, aged 45 years

However, the older adult volunteer shared an opposite viewpoint that the pull alarm worked better, as it could notify neighbors. In addition, hearing the alarm ring made her feel *it is useful*. She also specified its usefulness for different age groups. For older adults aged 60 to 70 years, she found the pull alarm to be more convenient. However, for those aged >70 years, she thought it was good for them to have SHS, as they might not be mobile enough to pull the cord.

One older adult had a previous bad experience with the pull alarm system. She accidentally pulled the cord and sounded the alarm, but no one had attended her for half an hour. This made her feel that even if she had installed SHS, it would not make much of a difference and no one would hear or attend her in case of an emergency. Although SHS is a completely different technology, the older adult still projected her bad experiences and distrust of the emergency systems onto it. Thus, she refused to continue using SHS.

The older adults also provided constructive suggestions to the researchers. They spoke about concerns regarding sensor locations where they felt uncomfortable, such as in the bathroom. In terms of more locations for emergency buttons, one older adult commented:

Because the pull alarm is installed in three rooms, you can pull it if you are near to any of them, for this emergency button [of SHS], you can only press the emergency button only when you are close to it. If the distance is too far, then you can’t reach it, most of the elderly feel dizzy, symptoms of stroke or heart attack, you can’t even walk.

E021, male, aged 85 years

Older adults who chose to adopt SHS prioritized its usefulness and necessity over its costs. An older adult shared:

If your product is good, even if it costs money, people will still install it, right? And if it’s bad, no one will install even if it’s free, right?

E030, male, aged 70 years

Therefore, affordability is not only linked to cost but also to perceived benefits, such as an increased sense of security, assurance, and safety. We found that understanding technology and perceived benefits and usefulness seem to be among the first factors older adults consider when deciding to adopt SHS technology, and these are intrinsic motivational factors.

### Theme 3: Concerns on Affordability

Most Singaporean older adults retire at 60 years of age and rely mainly on the Central Provident Fund for retirement, housing, and health care. We find that affordability concerns can be categorized into 2 categories: concerns about the consumption of electricity and subscription fees.

#### Subtheme 3.1: Consumption of Electricity

Concerns regarding the adoption of SHS include an increase in electricity consumption due to multiple sensors and electricity plugs. Eleven participants did not subscribe to SHS after the intervention, and one of the reasons was the perception that installing the system would increase their electricity expenses. Overall, 45% (5/11) of the participants indicated that an increase in electricity bills discouraged them from subscribing, 18% (2/11) were undecided, and 36% (4/11) indicated that an increase in electricity bills was not the reason why they did not subscribe. Participants presumed that the SHS would increase their consumption of electricity by 30%. In reality, the consumption of electricity has been measured by the SHS service provider to be much lower. However, for some older adults, extra electricity charges made them think twice about adopting SHS.

#### Subtheme 3.2: Concerns on Subscription Fees

The SHS monthly fee in Singapore dollars (SG) would be approximately SG $25 (US $18.2) based on its market price. This might not be much for a working person; however, there are a large number of retired older adults in Singapore. However, if older adults felt that SHS was beneficial and useful, they would be willing to save money to pay for it. An older adult shared:

It’s currently 25 [Singapore] dollars (US $18.2) a month, actually it’s ok, just consider it a dollar a day only mah, just eat a dollar less worth of food everyday lor [laughs]. It’s the same, isn’t it? Drink less coffee, spend less of everything. Still ok la. Because it is beneficial to us, especially us the elderly who live alone. It is considered a form of guarantee, a form of security for us, so it’s not bad lah.

E008, female, aged 70 years

Some older adults considered this amount reasonable; however, they were not sure whether others would find it affordable. Some felt that they could afford it if they could save some money elsewhere. One shared:

That is very reasonable for... the children give us and we can keep aside the money.

E040, female, aged 78 years

Some older adults (especially those who were healthier) did not think that the subscription fee was too large to pay. In the follow-up feedback, we asked participants who continued with the SHS subscription (n=31) to provide their estimate of an appropriate monthly subscription fee (in SG). Overall, 35% (11/31) of respondents indicated either between SG $10 (US $7.3) and SG $15 (US $11), 35% (11/31) of respondents indicated between SG $20 (US $14.5) and SG $25 (US $18.2), 19% (6/31) were undecided, and 10% (3/31) preferred to pay <SG $10 (US $7.3) or no fee at all (Multimedia Appendix 3).

As SHS were installed for a month, they became part of the older adults’ lives. Hence, some wanted to keep the SHS for themselves or for their spouses. They felt a need to continue with SHS either because of living alone (33/42, 79% lived alone) or medical issues (17/42, 40% had an average or less than average health status).

As most older adults aged ≥65 years are retired and have no income in Singapore, they rely heavily on their children for financial support, especially if they do not have sufficient savings. Thus, the relationship between older adults and their family caregivers (15/22, 68% of those with family support had adult children as caregivers) could adversely affect the affordability and subsequent adoption of SHS. If parents and children are in a good relationship, they can support their parents in paying for SHS. However, some older adults had issues with their children. One older adult complained:

My children should be the one paying, but they don’t want to help me. I told them—I have 4 daughters—it only requires each of you to fork out a few dollars every month. It’s just the cost of a bowl of noodles, am I right?... But if I’ll have to pay, there’s nothing I can do. I am not working, I have no money.

E014, female, aged 70 years

In addition, if older adults receive government subsidies, they might feel that they can afford SHS. However, financial support is only one form of support. Other forms of support from multiple stakeholders in the community are also required for technology adoption.

### Theme 4: Care Support From the Community

In this study, the importance of community support was highlighted. We noticed that older adults who could not rely on their family turned to the community for support. Some shared that they did not think it was necessary to install SHS, as they had no next of kin who would come to help:

I have nobody to rely on. I have a lot [of] nephews a lot of nieces but they have their own family to run.

E024, female, aged 64 years

Some believed that neighbors were more reliable than next of kin, who stayed far away. They expressed that in the case of an emergency, if they had to wait for help from their next of kin, they might die due to a delayed response. Thus, they would rather trust their neighbors.

One older adult shared that neighbors are often the best assistance in an emergency. Neighbors were suggested to be volunteer caregivers who could take care of each other:

The best [emergency response] is the nearby people come. [...] You get me? They nearest. Whoever. That’s why I tell [center manager], why don’t we have a committee that a group of people who want, volunteer, whoever want. [help to keep a lookout for each other during emergency] This comes from the heart. [...]

E024, female, aged 64 years

This close relationship among neighbors is not uncommon when all residents are aged ≥55 years, staying in the same HDB block. Some older adults felt very reassured with their neighbors who always *look for* him or her. Older adults believed that help from neighbors was necessary, especially during the weekends and nighttime. They found they must maintain the Kampung spirit (*Kampung*—village spirit refers to a sense of community and solidarity [23]). One older adult shared:

My neighbor will come look for me lah. They will say “how come I didn’t see you? [...] actually my floor the neighbor they are quite nice, When we don’t ... we don’t see...because ah I always sit outside the flat.”

E018, female, aged 61 years

During the intervention, we observed that some older adults preferred to have a volunteer, such as the center manager or a neighbor, as their emergency contact. We found 1 older adult who was willing to volunteer as a caregiver for several older adult participants during the intervention. This older adult volunteer felt that she takes care of her neighbors anyway every day and would want to help them in case of an emergency. She shared her thoughts that although family members can be predefined as contact persons in case of an emergency, she did not think they could come immediately if they stayed far from their parents. She shared:

Neighbors are more important, you know? My door is always open when I'm in, when close means nobody in. So, the neighbor always you know, they they... ah. They will know of my existence.

E018, female, aged 61 years

Support from center managers is also important for encouraging older adults to adopt SHS. We found that when SHS were recommended by the center manager, older adults were more likely to accept them, especially if they had a good relationship with the center manager.

In addition, some older adults found the SHS service provider to be very friendly and helpful and felt reassured that the SHS could be well maintained by the service provider. Some shared that the service provider had said:

This one [increase of electricity price] compared to your life—your life it’s more important.

E024, female, aged 64 years

The older adults shared the same viewpoint and thus felt encouraged by the SHS service provider to continue using the SHS.

On the basis of these findings, we developed an older adult–centric decision-making model involving 5 layered factors (Figure 2), ranging from intrinsic *motivated factors* such as understanding the technology and perceiving its benefits and usefulness to more *extrinsic motivated factors* such as affordability and support from multiple stakeholders in the community, which could encourage older adults toward a decision to adopt SHS technology.

## Discussion

### Principal Findings

Our findings show that multiple factors are involved in the decision-making process toward the adoption of SHS by older adults, and we acknowledge that not all factors could be exhaustively captured in a single study. However, we find that the insights gained from this study and the proposed layered factors involved in the decision-making process could be used to guide more informed awareness when considering the adoption of technology by older adults.

Although adult children and family members feel empowered by SHS to monitor their parents and be alerted when an emergency occurs, older adults are often left misinformed about the technology. Adult children often feel confident in their ability to persuade their parents to adopt the given technology [24]. However, we found from our study that older adults need not only persuasion or reassurance from their family but also empowerment to understand the technology, thereby enabling them to make informed decisions through adequate knowledge and appreciation of the benefits of the technology [25,26].

Moreover, in contrast to the prevalent misconceptions, older adults show more positive than negative attitudes toward technology. Older adults’ desire to learn, ability to understand, and willingness to use new technology have often been underestimated [27]. From our study, we find that there exists a gap between the expected and the actual understanding of technology. Older adults lacked an introduction to, as well as sufficient, information about SHS. Training materials, with the aim of teaching older adults the functionality of the technology, should be provided in an accessible form, such as simple and concise user manuals, verbal introduction by the SHS service provider, short testimonial videos, or visuals in posters. Older adults’ questions and concerns could be addressed as frequently asked questions posed by older adults. These training materials and educational concepts focused on older adults as the main users of the technology could help improve vendor outreach and ultimately drive new government policy.

From our findings, we identified human needs for trust, privacy, and personal autonomy when older adults considered adopting SHS. Human values are the guiding principles of what is considered important in people’s lives and often go beyond financial considerations [28]. People’s choices or engagement with technology depend on how technology can support them in maintaining their values [29]. These human values are important to be identified for a better design and development of assistive technology.

In Singapore, there is a general sense of public trust in the government as it places increasing emphasis on citizen well-being and public services. Citizens can benefit from outcomes translated from policy implementation [30]. Our study was facilitated by pre-existing trust and long-standing relationships between older adults and the research team, SAC center managers, and SHS service providers. There had been a long-standing research collaboration between the research team at NTU and SAC, including several research projects and community engagement events; for example, the Singapore Intergenerational National Games [31]. In addition, the pre-existing relationship with SAC center managers was critical in the recruitment of participants for the study. We found that older adults valued the center manager’s opinions and trusted their recommendations to use the SHS. Furthermore, there had been a program launched at the SAC together with the SHS service provider to install SHS at the SAC; thus, the older adults were somewhat familiar with the SHS service provider and SHS technology [32]. We observed that older adults’ positive attitudes toward the SHS service providers could lead to greater user satisfaction and continued adoption of SHS.

Furthermore, we found that the older adults in our study trusted the SHS service provider and SAC center manager in handling their private data. One study suggests that users trusted the company providing SHS and felt that they were not worthwhile targets for privacy breaches [33]. As trust in entities that collect data is related to the need for users to have control over their own data [29], this was a critical factor in the use and adoption of SHS in our study.

Although older adults might trust the SHS service provider with their private data, they might not want to be constantly monitored and have all their movement data shared with emergency contacts. Privacy concerns have been recognized as barriers to adopting sensor-based technologies that use video surveillance systems [34]. One study found that older adults and their adult children perceived privacy as the most-cited concern [24]. In our study, the SHS system provided movement data of older adults to their adult children or caregivers, which could become a potential issue of privacy and personal autonomy. For some, sharing personal information with people with whom they are close to may make them feel safer; however, there does not seem to be a universal consensus regarding this [33]. Older adults’ relationships with their adult children could be very different from case to case, and for various reasons, the amount of personal information they may be willing to share could differ. To address this potential issue, we suggest that older adults be empowered to predefine the person, duration, and range of personal data they wish to share to maintain their autonomy.

Technology is becoming increasingly ubiquitous, and older adults may find it difficult to identify perceived benefits, especially when the user does not directly interact with the technology, as in the case of SHS, unless there is an emergency. Many older adults would be unfamiliar with this rather new technology, as SHS was only introduced to Singapore less than 10 years ago when HDB piloted the Smart Elderly Monitoring and Alert System in HDB flats where older adult residents resided in 2014 [35]. A novelty of SHS is its discreet monitoring of movement, unlike well-known camera surveillance systems that capture images and voices. On the one hand, this rather passive use of SHS makes its acceptance easier for older adults, as it is considered very nonintrusive and can be well integrated into daily lives. On the other hand, we find that this novelty makes it challenging for older adults to fully grasp the benefits of SHS. It could lead to the feeling of “it makes no difference for me” or “it is useless.” However, this could indicate a deeper human need for personal autonomy.

In this study, we observed that older adults shared common personal autonomy values. On the one hand, older adults would rather trouble their friends or neighbors when they needed help, which, in the Asian context, is considered an integral part of friendship. In being able to lend a helping hand to others, older adults felt validated that they were still useful and capable. This helps to maintain the friendship network. On the other hand, older adults tend to be more optimistic about their own future than someone else’s at a very old age [36]. Horton [37] found that older adults had the impression that other people would benefit from assistive technology but not themselves. In our study, particularly those who did not adopt the SHS after the intervention expressed that they were capable of handling most situations and downplayed the severity of situations they might encounter. They held on to their personal autonomy by showing little or no interest in SHS. Many studies have accounted for this attitude of older adults resisting assistive technology because it associates them with the negative context of becoming old [8].

We found that a user-centric design involving older adults is essential for the successful adoption of technology [38,39]. System designers should strive to understand the requirements of older adults, incorporate their feedback, and adapt technology to meet age-related needs, such as changes in abilities, health status, living arrangements, and family structures [40]. During our study, older adults provided feedback on the design of the SHS system, thereby demonstrating their active wish to participate in the improvement and design of SHS. Thus, we found that older adults should be more involved in the user-centric design of SHS technology based on human values.

For example, during the study, we identified the need to rely on neighbors and fellow older adults in the community rather than on their children who live far away. Thus, we made a simple change to the intervention design by including an older adult volunteer as emergency contact for some older adult participants in the study. This shows how the needs of older adults can be incorporated into the implementation of SHS, which could eventually lead to a higher adoption rate of the technology. We also found that older adults expressed the need to hear an emergency alarm when it was triggered using the pull lever in the old system. The sounding of an alarm not only gave the older adults feedback and reassurance that the call for help was successfully made but also that the loud noise would alert neighbors who they trusted would quickly come to help them. In contrast, the older adults were unsure how they would know if the SHS alarm was successfully triggered in the case of an emergency, as no alarm was sounded. This example shows, on the one hand, how technology could potentially disrupt existing relationships and support structures and be detrimental to the community spirit of helping one another. On the other hand, these findings also present an opportunity for technology to be adapted to the needs of older adults. The sensors need not only trigger a silently sent alarm to the emergency contact but they can also be redesigned to additionally sound an alarm. This would preserve the existing emergency response structures in the community and among neighbors and still have all the advantages of the new system.

Furthermore, we identified the need to develop custom technology solutions for different aging societies [41]. In our study, the uniqueness of the Singapore setting [42] highlights the importance of a support ecosystem involving multiple stakeholders in the community, ranging from family to neighbors, center managers, and SHS service providers, in older adults’ decision-making processes to adopt technology. Although the engineering teams focused on prototype and algorithm development, the medical science teams concentrated on outcome research. The convergence of medicine and informatics could lead to the development of new interdisciplinary research models and new assistive products for the care of older adults.

### Limitations

Although we identified the need for financial support when older adults consider the adoption of SHS, in this study, we could not further investigate the impact of cost on the decision to adopt technology, as study participants were offered a 2-year free subscription if they wished to continue using the SHS. Thus, we would need to investigate whether the introduction of a subscription fee has an effect on the number of older adults who continue or who subsequently drop the SHS subscription.

The inclusion criteria were subjectively assessed, and this could be improved by using a more objective method, such as a questionnaire, or performing a set of tasks to assess this. In addition, cognitive ability was not assessed, which could play a role in the willingness and ability to adopt technology.

We acknowledge that our findings are specific to the context and unique structures surrounding living and caring for older adults in Singapore. Older adults who choose to live in an SAC are generally more open to trust center managers and their neighbors and are more willing to be helped by others. They also sought to share information with others and integrate it into the community. For example, some older adults share home-cooked meals and often participate in activities organized by the SAC. As such, our findings may not be generalizable to other countries with different circumstances. Nevertheless, our insights could serve as inspiration for potential solutions.

### Conclusions

This study combined quantitative and qualitative methods to explore the factors and influences of the decision-making process toward the adoption of SHS technology by older adults in Singapore. SHS were installed in the homes of 42 older adults for a period of 4 to 5 weeks. Our findings show that both intrinsic and extrinsic factors are involved in the acceptance and adoption of SHS technology. We found that training and a strong support ecosystem could empower older adults in their decisions to adopt technology.

We also identified human values, such as trust, privacy, and personal autonomy, as important factors in influencing older adults’ choices of engagement with SHS. Our study was facilitated by long-built trust between older adults and multiple stakeholders, which was established over the years through various activities held by SACs, programs organized by NTU, and interactions between older adults and the research team.

In the future, besides providing adequate training for older adults to understand the technology they are to use, we advise their involvement of older adults in the design process to build user-centric assistive technology. We find it important to consider the integration of human values in technological solutions and their adaptation to the needs of older adults. In addition, these systems should be evaluated using qualitative methods to explore lived experiences with the technology.

## Appendix

Multimedia Appendix 1 Interview questions.

Multimedia Appendix 2 Coding scheme, sample quotes, and mappings to themes and subthemes.

Multimedia Appendix 3 Follow-up feedback questionnaire with responses.

## Abbreviations

AHE: Adventist Home for the Elders
NTU: Nanyang Technological University
SAC: senior activity center
SG: Singapore dollars
SHS: smart home sensors
TAM: Technology Acceptance Model

## References

[1] (2016). I feel young in my Singapore! Action plan for successful ageing. Ministry of Health, Singapore.

[2] Agboola S, Golas S, Fischer N, Nikolova-Simons M, Op den Buijs J, Schertzer L, Kvedar J, Jethwani K (2017). Healthcare utilization in older patients using personal emergency response systems: an analysis of electronic health records and medical alert data : brief description: a longitudinal retrospective analyses of healthcare utilization rates in older patients using Personal Emergency Response Systems from 2011 to 2015. BMC Health Serv Res.

[3] (2020). HDB Smart Enabled Home. Housing & Development Board.

[4] Dibner AS (1990). Personal emergency response systems: communication technology aids elderly and their families. J Appl Gerontol.

[5] McKenna AC, Kloseck M, Crilly R, Polgar J (2015). Purchasing and Using Personal Emergency Response Systems (PERS): how decisions are made by community-dwelling seniors in Canada. BMC Geriatr.

[6] Heinbüchner B, Hautzinger M, Becker C, Pfeiffer K (2010). Satisfaction and use of personal emergency response systems. Z Gerontol Geriatr.

[7] Ehrenhard M, Kijl B, Nieuwenhuis L (2014). Market adoption barriers of multi-stakeholder technology: smart homes for the aging population. Technol Forecast Soc Change.

[8] Astell A, McGrath C, Dove E (2020). ‘That's for old so and so's!’: does identity influence older adults’ technology adoption decisions?. Ageing Soc.

[9] Chen K, Chan AH (2013). Use or non-use of gerontechnology--a qualitative study. Int J Environ Res Public Health.

[10] Melenhorst AS, Rogers WA, Caylor EC (2001). The use of communication technologies by older adults: exploring the benefits from the user's perspective. Proc Hum Factors Ergon Soc Annu Meet.

[11] Davis FD (1989). Perceived usefulness, perceived ease of use, and user acceptance of information technology. MIS Q.

[12] Pan S, Jordan-Marsh M (2010). Internet use intention and adoption among Chinese older adults: from the expanded technology acceptance model perspective. Comput Human Behav.

[13] Pal D, Funilkul S, Charoenkitkarn N, Kanthamanon P (2018). Internet-of-things and smart homes for elderly healthcare: an end user perspective. IEEE Access.

[14] Russell TG, Gillespie N, Hartley N, Theodoros D, Hill A, Gray L (2015). Exploring the predictors of home telehealth uptake by elderly Australian healthcare consumers. J Telemed Telecare.

[15] Venkatesh V, Morris MG, Davis GB, Davis FD (2003). User acceptance of information technology: toward a unified view. MIS Q.

[16] Davenport RD, Mann W, Lutz B (2012). How older adults make decisions regarding smart technology: an ethnographic approach. Assist Technol.

[17] Emmel N, Emmel N (2013). Purposeful sampling. Sampling and Choosing Cases in Qualitative Research: A Realist Approach.

[18] Sekhon M, Cartwright M, Francis JJ (2018). Acceptability of health care interventions: a theoretical framework and proposed research agenda. Br J Health Psychol.

[19] Fereday J, Muir-Cochrane E (2006). Demonstrating rigor using thematic analysis: a hybrid approach of inductive and deductive coding and theme development. Int J Qual Methods.

[20] Boyatzis RE (1998). Transforming Qualitative Information: Thematic Analysis and Code Development.

[21] Nili A, Tate M, Barros A, Johnstone D (2020). An approach for selecting and using a method of inter-coder reliability in information management research. Int J Inf Manag.

[22] Croff RL, Witter Iv P, Walker ML, Francois E, Quinn C, Riley TC, Sharma NF, Kaye JA (2019). Things are changing so fast: integrative technology for preserving cognitive health and community history. Gerontologist.

[23] Blast from the past. National Archives of Singapore.

[24] Berridge C, Wetle TF (2020). Why older adults and their children disagree about in-home surveillance technology, sensors, and tracking. Gerontologist.

[25] Le Dantec CA, Poole ES, Wyche SP (2009). Values as lived experience: evolving value sensitive design in support of value discovery. Proceedings of the SIGCHI Conference on Human Factors in Computing Systems.

[26] Sellen A, Rogers Y, Harper R, Rodden T (2009). Reflecting human values in the digital age. Commun ACM.

[27] Mitzner TL, Boron JB, Fausset CB, Adams AE, Charness N, Czaja SJ, Dijkstra K, Fisk AD, Rogers WA, Sharit J (2010). Older adults talk technology: technology usage and attitudes. Comput Human Behav.

[28] Miller JK, Friedman B, Jancke G, Gill B (2007). Value tensions in design: the value sensitive design, development, and appropriation of a corporation's groupware system. Proceedings of the 2007 International ACM Conference on Supporting Group Work.

[29] Worthy P, Matthews B, Viller S (2016). Trust me: doubts and concerns living with the Internet of Things. Proceedings of the 2016 ACM Conference on Designing Interactive Systems.

[30] Chan D (2018). Public Trust in Singapore.

[31] Centre for Healthy and Sustainable Cities (CHESS) Singapore Intergenerational National Game. Nanyang Technological University.

[32] Teng A (2016). Sensor system to help keep seniors safe at home. The Straits Times.

[33] Zeng E, Mare S, Roesner F (2017). End user security and privacy concerns with smart homes. Proceedings of the 13th Symposium on Usable Privacy and Security.

[34] Demiris G, Oliver DP, Giger J, Skubic M, Rantz M (2009). Older adults' privacy considerations for vision based recognition methods of eldercare applications. Technol Health Care.

[35] (2014). Smart Nation: Vision. Prime Minister's Office Singapore.

[36] Durbin KA, Barber SJ, Brown M, Mather M (2019). Optimism for the future in younger and older adults. J Gerontol B Psychol Sci Soc Sci.

[37] Horton K (2008). Falls in older people: the place of telemonitoring in rehabilitation. J Rehabil Res Dev.

[38] Czaja SJ, Lee CC (2006). The impact of aging on access to technology. Univ Access Inf Soc.

[39] Tiersen F, Batey P, Harrison MJ, Naar L, Serban AI, Daniels SJ, Calvo RA (2021). Smart home sensing and monitoring in households with dementia: user-centered design approach. JMIR Aging.

[40] Czaja SJ, Boot WR, Charness N, Rogers WA (2019). Designing for Older Adults: Principles and Creative Human Factors Approaches. 3rd edition.

[41] Sapci AH, Sapci HA (2019). Innovative assisted living tools, remote monitoring technologies, artificial intelligence-driven solutions, and robotic systems for aging societies: systematic review. JMIR Aging.

[42] Ho EL, Huang S (2018). Care Where You Are: Enabling Singaporeans to Age Well in the Country. Straits Times Press.

