# Supplementary material for: Decision-making Factors Toward the Adoption of Smart Home Sensors by Older Adults in Singapore: Mixed Methods Study
Source: JMIR Aging. 2022 Jun 24;5(2):e34239. doi: 10.2196/34239 (PMC9270706; doi:10.2196/34239)
Supplement: Multimedia Appendix 1 [file aging_v5i2e34239_app1.docx]

Interview questions.

As a guideline to the interviewers, interview questions for the older adults, family caregivers and center managers at Adventist Home for the Elders (AHE) were prepared. Service mentioned in interviews referred to smart home sensors (SHS). Participants were aware of this when they were interviewed. When interviewers conducted the interviews, they provided examples such as falling at home and feeling unsecure when alone to illustrate what was meant by problem. Most interviews were conducted in Mandarin, and in Mandarin, problem is synonymous with concern. In addition, the interviewers explained the meaning of terms according to the current context of the participants.

| **Interview questions (for older adults)** |
| --- |
| 1. How do you feel about the current service (SHS)? Do you find it useful? |
| 1. How did it solve your problem after the installation of home sensors? |
| 1. Do you feel reassured knowing that there is the sensor to inform your next of kin in the event you have any accident? |
| 1. What does your next of kin feel about this service (SHS)? Do they think it is a good service (SHS)? |
| 1. Were there any instances when you felt uncomfortable because of the device? Why so? |
| 1. How did this service (SHS) benefit you? Would you please share some details with us? |
| 1. If you are given the choice to continue this service (SHS) for the next 2 years, would you?   If not, why not? |
| 1. How much should the subscription fee be in your opinion? |
| 1. What suggestions do you have to improve this service (SHS)? |
| **Interview questions (for family caregivers)** |
| 1. How do you feel about the current service (SHS)? Do you find it useful for you? How much do you find that it helps you as a caregiver? |
| 1. Did it solve your problem after the installation of home sensors? Did it reduce your burden of care? |
| 1. How did it benefit you and your family member? Would you please share some details with us? |
| 1. What suggestions do you have to improve this service (SHS)? |
| **Interview questions (for center managers at AHE)** |
| 1. Do you think this service is needed for older adults who stay here? How useful do you think it is for them? |
| 1. What were the challenges you faced when you were explaining this service to older adults? |
| 1. What suggestions do you have to improve this service (SHS)? |
