# Supplementary material for: Decision-making Factors Toward the Adoption of Smart Home Sensors by Older Adults in Singapore: Mixed Methods Study
Source: JMIR Aging. 2022 Jun 24;5(2):e34239. doi: 10.2196/34239 (PMC9270706; doi:10.2196/34239)
Supplement: Multimedia Appendix 2 [file aging_v5i2e34239_app2.docx]

Coding scheme, sample quotes and mappings to themes and sub-themes.

The coding scheme defined the codes and subcodes to be used for coding the transcripts of the interviews. The code categories and subcodes were mapped to themes and subthemes. Sample quotes are excerpts from the coded transcripts to illustrate the mapping of code categories and subcodes to the themes.

| **Code categories** | **Subcode** | **Sample quotes** | **Themes** |
| --- | --- | --- | --- |
| **Personal perception on general services** | Service satisfaction or dissatisfaction | Older adult: “[…] this app is very useful already. So, I know he’s at home right, so what I do is okay I can check what is his last activity. [...] Any emergency would have to press that button. So if any water leakage anything also I will know. So this is very useful.”  Older adult: “I think this sensor will be better (than the emergency pull cord). This one if you are not at the place there, how you going to pull? If you really cannot move, you see cannot move, like the uncle that fall down in the kitchen, the toilet there ah [...] He also cannot pull the thing, he already slid down. [...] It's too far.” | Subtheme 2.1: psychological benefits  Subtheme 2.2: preference of SHS over existing monitoring systems |
|  | Understanding of service | Older adult: “Right, it only senses. Some people don’t understand. They thought it was an infringement of their privacy, but actually it is not. I told them it is not, so I feel that this sensor is very good. [....]”  Interviewer: “Do you feel a little uneasy having a technological device like this installed in your home?”  Older adult: “No ah.” | Theme1: understanding SHS functionality |
|  | Service reliance | Interviewer: “Will you keep SHS after 2-year free package?”  Older adult: “I will try and ah keep it ah. [...] It's a necessity.” | Subtheme2.1: psychological benefits |
|  | Service affordability | Interviewer: “Is it affordable for you?”  Older adult: “I think it should be okay la because as I told [SHS service provider] I’m still looking for part-time job.”  Older adult: “[Money is] a factor, you know why? Old people no income what, that no income.” | Subtheme 3.2 concerns on subscription fees |
|  | Usefulness of service: for self or for others | Interviewer: “I’d like to talk to you about the sensors installed in your house. Do you think it is useful for you?”  Older adult: *“*Very good! My goddaughter says, I know you have overslept by a few hours (Laughs) Because I don’t have children mah. I have a goddaughter, so she downloaded the app. I asked her what it was like, and she said, ‘I know you have slept for six hours’.”  Older adult: “But then that's for the very- I think maybe for the very very old people lah”  Interviewer: “For- Oh! Ok ok. So, you think that it is more useful for those-“  (Overlap)  Older adult: “Because you know why! Think for the sick will be better. I don't think I need it.”  Interviewer: “you can take care of yourself, and you think, if let's say in any case of emergency, you just need a phone to call someone?”  (Overlap)  Older adult: “Ya, I need- I need a phone. I just need a phone.” | Theme2: perceived benefits and usefulness of SHS:  Subtheme2.1: psychological benefits |
| **Previous experiences on services** |  | Older adult: “Honestly, this pull cord is not feasible. Because…when you fall down when you are home alone, how are you going to crawl all the way there to pull the cord. The emergency pull cord works too, but if there’s only one (pull cord) in the house, how is someone who falls over there supposed to come all the way here to pull the pull cord? Let me tell you, the emergency pull cords are designed wrongly. You won’t be able to reach it if you’re sitting down or sitting at somewhere low. So it’s better to have something that you can press.” | Subtheme 2.2: preference of SHS over existing monitoring systems |
| **Motivation** | Motivation or reason to participate in service | Older adult: “Ah all these I already- I know because my neighbour the other time like in early 2018 [possibly Feasibility study], I think she already get I think like oh okay this is good actually [...] She already have it so I know what is actually happening (Laughter) [...] I’m aware of it long time ago.”  Older adult: “I want to try. This is not bad la. It has many benefits, not bad. For the elderly who have difficulty walking, it is very useful to them. Besides, every elderly has some medical condition of his/her own, right? Look at me, my heart is not that good, so installing this (smart home sensor) is very helpful.” | Theme 1: understanding SHS functionality and Theme 4: support from the community (recommendation from neighbours)  Theme 2: perceived benefits and usefulness of SHS |
|  | Motivation or reason to not participate in service | Interviewer: “Will you be interested in a two-year package?”  Older adult: “Not yet. I need to see what the two-year package is about. [...] I have to see what the sensors are like in the next two years. Say there’s an emergency at home a year later. I would like to know who it is who will respond to my emergency and come and render me help.” | Theme 1: understanding SHS functionality and  Theme 4: support from the community |
| **Personal information** | Memory and medication routine | Interviewer: “So the installation where they have this feature when you go outside… like a key or something when you go out?”  Older adult: “Ya when he goes out, he carry that keychain and I know he goes out. [...] He has dementia… Got a few times he go out I won’t know. I… but nowadays he don’t go out la. Here he stay here. Last time, he went out and about 4-5 times, he didn’t come back and have to go and hunt and then I tell ‘Lord, you bring him back’.” |  |
|  | Dependence on self or others | Older adult: “She (daughter) said ‘$25 is ok, don’t worry I will pay’. Well as long as she says ok. If she doesn’t pay then you all can take it back.” | Sub-Theme 3.2: concerns on subscription fees |
|  | Living arrangement | Older adult: “My neighbour will come look for me lah. They will say ‘how come I didn't see you..?’”  Interviewer: “They will peep at you, like ‘are you alright in there?’”  (Overlap)  Older adult: “Ya, ya, ya, actually my floor the neighbor they are quite nice, When we don’t … we don't see...because ah I always sit outside the flat”  Interviewer: “Oh, that's nice. You- oh so you always open your door”  Older adult: “Yah, my door is always open when I'm in, when close means nobody in. So, the neighbor always you know. They will know of my existence.” | Theme 4: support from the community |
|  | Financial arrangement and financial worries | Interviewer: “So let’s say this sensor ever it charge on your bills, What’s the maximum in dollars?”  Older adult: “I don’t think so cause I feel my bill is still as usual but I don’t really care I just pay without thinking” | Sub-Theme 3.2: concerns on subscription fees |
| **Perception towards professionals or service** | Towards professionals (center managers or nutritionists or nurses or SHS service providers) | Interviewer: “I’m so sorry ya. But I really hope that any point of time if let’s say any sensor related, please let us know.”  Older adult: “Ya I will! [SHS service provider staff’s name] will know what, everything because [SHS service provider staff’s name] will see the system and check. I think he will give monthly check or whatever check ah [SHS service provider staff’s name].” |  |
|  | Towards service | Interviewer: “[...] That’s great. So for that 5 weeks do you feel like you were intruded, your life is intruded or...”  Older adult: “Because it’s all by written or like captured like … they show me all the graph la.”  Interviewer: “So you totally trust this app and the sensor?”  Older adult: (Laughter) “yah I trust I trust.” | Theme 1: understanding SHS functionality |
| **Issues with hardware design** | Issues with hardware design | Older adult: “[For] the monitor (sensors), I feel like there are a lot of gadgets here and there, you know. There is the monitor (sensor), and we are told not to switch it off. Sometimes my domestic helper comes (to clean the house), you know. There are a bit too many wiring (on the floor) I think.” |  |
| **Personal suggestions on services** | Suggestions for service | Interviewer: “So for your suggestion about installing emergency panic buttons at a lower height – how many panic buttons do you think is enough?”  Older adult: “Like there must be one in the toilet, right? Then one in the living room. It’s necessary to install one in the room too, by the bed. Two in fact – one at the head and one at the foot of the bed. So that the person can press the button to alert the others when he or she had a fall. [...] There must be one in the kitchen.”  Older adult: “Ah…but it would be better if they teach us how to…they always install in a hurry and they did not…they did not like…take time to teach us how to…like explain to us what this (the sensor) is for, what that is for…right? [...] Ah and also, they only told us these sensors are for safety. They did not…touch on…did not tell us what the overall service is like. Interviewer: “Hmm, so you mean it will be better if they can be more in-depth, more meticulous in explaining, right?”  Older adult: “Ah, yes. Or they could have a…like a seminar to brief us on this too.”  Interviewer: “Ah I see.”  Older adult: “Like (explaining to interviewer), what is the purpose of installing one in the bedroom, why one in the kitchen and one in the washroom etc. Ah…like they can have a presentation at the center downstairs, project the sensor onto the screen and tell us ‘ah the reason to have one sensor installed in the kitchen is so that it can detect leaked gas’ or something along those lines.” | Theme 1: understanding SHS functionality. The eagerness to understand functionality, and older adults want to be empowered with knowledge. |
