# Supplementary material for: Decision-making Factors Toward the Adoption of Smart Home Sensors by Older Adults in Singapore: Mixed Methods Study
Source: JMIR Aging. 2022 Jun 24;5(2):e34239. doi: 10.2196/34239 (PMC9270706; doi:10.2196/34239)
Supplement: Multimedia Appendix 3 [file aging_v5i2e34239_app3.docx]

Follow-up feedback questionnaire.

A self-report questionnaire using a 5-point Likert scale, designed to elicit feedback after intervention from participants of the study regarding their reasons for adopting or not adopting smart home sensors. Two open-ended questions are asked regarding the subscription fee and intention to subscribe after the 2-year free subscription. Responses from participants are summarized.

| These questions ask about your intentions and decision to subscribe to Commercial Smart Living Solution by ConnectedLife. Please indicate how true the sentences are to you. | | | | | | | | |
| --- | --- | --- | --- | --- | --- | --- | --- | --- |
| The reason I decided to subscribe to Smart Living Solution is because: | | 1-Not Very True | | 2-Not True | 3-Undecided | | 4-True | 5-Very True |
| 1. It is free for the first 2 years of subscription. | | 9 | | 3 | 2 | | 7 | 10 |
| 1. I live alone | | 1 | | 1 | 3 | | 13 | 13 |
| 1. It provides me with reassurance that I will get help in a timely manner | | 0 | | 1 | 3 | | 11 | 16 |
| 1. I can contact someone for immediate help in times of emergency. | | 0 | | 1 | 1 | | 16 | 13 |
| 1. My family members will be contacted immediately in times of emergency | | 0 | | 2 | 1 | | 16 | 12 |
| 1. My friends and neighbours have also subscribed | | 9 | | 3 | 4 | | 10 | 5 |
|  | | | | | | | | |
| The reason I decided NOT to subscribe to Smart Living Solution is because: | | 1-Not Very True | | 2-Not True | 3-Undecided | | 4-True | 5-Very True |
| 1. I do not understand how the system works | | 5 | | 1 | 1 | | 4 | 0 |
| 1. I do not see a benefit of having the system installed | | 1 | | 1 | 0 | | 4 | 5 |
| 1. I do not want to bother my family caregivers | | 3 | | 2 | 2 | | 1 | 3 |
| 1. I am rarely at home | | 3 | | 2 | 2 | | 1 | 3 |
| 1. It will increase my electricity expenses | | 4 | | 0 | 2 | | 1 | 4 |
| 1. I would need to pay for subscription fees after the 2-year free subscription | | 5 | | 1 | 2 | | 1 | 2 |
| **Open-ended questions** | | | | | | | | |
|  | Summary of Responses | | Number of older adults who subscribed (n=31) | | | Number of older adults who did not subscribe (n=11) | | |
| 1. After the 2-year free subscription is over, how much do you think you are willing to pay for subsequent subscription fees? | $20-$25 per month | | 11 (35%) | | | 0 (0%) | | |
|  | $10-$15 per month | | 11 (35%) | | | 0 (0%) | | |
|  | $5 or less per month | | 2 (7%) | | | 1 (9%) | | |
|  | Unsure or undecided | | 5 (16%) | | | 3 (27%) | | |
|  | Unwilling to pay | | 1 (3%) | | | 0 (0%) | | |
|  | Fear of being monitored | | 0 (0%) | | | 1 (9%) | | |
|  | Not interested | | 0 (0%) | | | 1 (9%) | | |
|  | No comment or NA | | 1 (3%) | | | 5 (45%) | | |
| 1. After the 2-year free subscription is over, how long do you think you will subscribe to the service? Please explain why. | As long as possible | | 6 (19%) | | | 0 (0%) | | |
|  | As long as residing at AHE | | 7 (23%) | | | 0 (0%) | | |
|  | As long as financially possible | | 6 (19%) | | | 0 (0%) | | |
|  | As long as service is good | | 1 (3%) | | | 0 (0%) | | |
|  | 3 years | | 1 (3%) | | | 0 (0%) | | |
|  | Unsure or undecided | | 9 (29%) | | | 2 (18%) | | |
|  | Unwilling to subscribe further | | 1 (3%) | | | 3 (27%) | | |
|  | No comment or NA | | 0 (0%) | | | 6 (55%) | | |
